# Ethyl 4-{[1-(2,4-dichloro­benz­yl)-1*H*-1,2,3-triazol-4-yl]meth­oxy}-8-(trifluoro­meth­yl)quinoline-3-carboxyl­ate

**DOI:** 10.1107/S1600536812039633

**Published:** 2012-09-26

**Authors:** Hoong-Kun Fun, Chin Wei Ooi, B. Garudachari, Arun M. Isloor, Suraya A. Rashid

**Affiliations:** a X-ray Crystallography Unit, School of Physics, Universiti Sains Malaysia, 11800 USM, Penang, Malaysia; b Department of Pharmaceutical Chemistry, College of Pharmacy, King Saud University, PO Box 2457, Riyadh 11451, Saudi Arabia; c Medicinal Chemistry Laboratory, Department of Chemistry, National Institute of Technology–Karnataka, Surathkal, Mangalore 575 025, India; d Department of Chemical and Environmental Engineering, Faculty of Engineering, Universiti Putra Malaysia, 43400 UPM SERDANG, Selangor, Malaysia

## Abstract

In the title compound, C_23_H_17_Cl_2_F_3_N_4_O_3_, the triazole ring makes dihedral angles of 50.27 (6) and 82.78 (7)° with the quinoline ring system and the dichloro-substituted benzene ring. The dihedral angle between the quinoline and dichloro-substituted benzene rings is 38.17 (4)°. In the crystal, mol­ecules are linked *via* C—H⋯N, C—H⋯F and C—H⋯O hydrogen bonds into a three-dimensional network. The crystal is further consolidated by C—H⋯π contacts to the triazole ring and inversion-related π–π inter­actions between the benzene and pyridine rings of quinoline systems [centroid–centroid distance = 3.7037 (7) Å].

## Related literature

For background and the biological activity of quinoline derivatives, see: Bi *et al.* (2004 ▶); He *et al.* (2005 ▶); Holla *et al.* (2006 ▶); Isloor *et al.* (2000 ▶, 2009 ▶); Vijesh *et al.* (2010 ▶). For bond-length data, see: Allen *et al.* (1987 ▶). For the stability of the temperature controller used for data collection, see: Cosier & Glazer (1986 ▶).
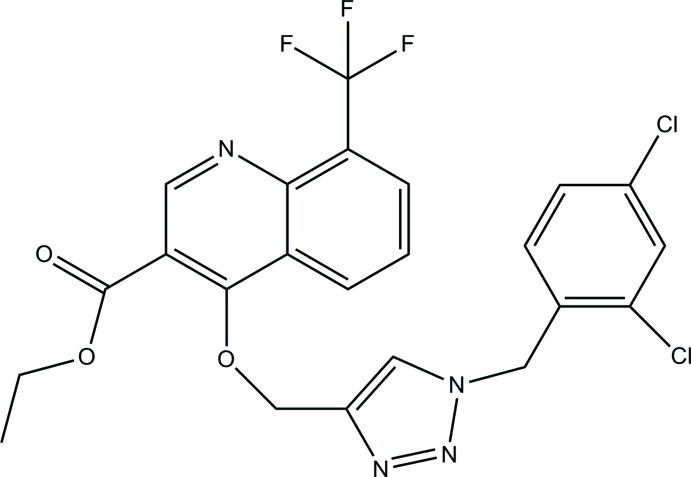


## Experimental

### 

#### Crystal data

C_23_H_17_Cl_2_F_3_N_4_O_3_
*M* *_r_* = 525.31
Monoclinic, 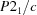

*a* = 10.0414 (6) Å
*b* = 18.3997 (11) Å
*c* = 15.5456 (7) Å
β = 128.559 (2)°
*V* = 2246.0 (2) Å^3^
*Z* = 4
Mo *K*α radiation
μ = 0.35 mm^−1^
*T* = 100 K
0.32 × 0.31 × 0.17 mm

#### Data collection

Bruker APEX DUO CCD area-detector diffractometer
Absorption correction: multi-scan (*SADABS*; Bruker, 2009 ▶) *T*_min_ = 0.896, *T*_max_ = 0.942
28920 measured reflections
8173 independent reflections
6633 reflections with *I* > 2σ(*I*)
*R*_int_ = 0.028

#### Refinement

*R*[*F* ^2^ > 2σ(*F* ^2^)] = 0.035
*wR*(*F* ^2^) = 0.102
*S* = 1.03
8173 reflections
317 parameters
H-atom parameters constrained
Δρ_max_ = 0.49 e Å^−3^
Δρ_min_ = −0.27 e Å^−3^

### 

Data collection: *APEX2* (Bruker, 2009 ▶); cell refinement: *SAINT* (Bruker, 2009 ▶); data reduction: *SAINT*; program(s) used to solve structure: *SHELXTL* (Sheldrick, 2008 ▶); program(s) used to refine structure: *SHELXTL*; molecular graphics: *SHELXTL*; software used to prepare material for publication: *SHELXTL* and *PLATON* (Spek, 2009 ▶).

## Supplementary Material

Crystal structure: contains datablock(s) global, I. DOI: 10.1107/S1600536812039633/sj5262sup1.cif

Structure factors: contains datablock(s) I. DOI: 10.1107/S1600536812039633/sj5262Isup2.hkl

Supplementary material file. DOI: 10.1107/S1600536812039633/sj5262Isup3.cml

Additional supplementary materials:  crystallographic information; 3D view; checkCIF report

## Figures and Tables

**Table 1:** Hydrogen-bond geometry (Å, °) *Cg*1 is the centroid of the N1–N3/C8/C9 triazole ring

*D*—H⋯*A*	*D*—H	H⋯*A*	*D*⋯*A*	*D*—H⋯*A*
C5—H5*A*⋯N3^i^	0.93	2.62	3.3330 (14)	134
C7—H7*A*⋯F1^ii^	0.97	2.46	3.1712 (15)	130
C8—H8*A*⋯O2^iii^	0.93	2.25	3.0183 (18)	139
C2—H2*A*⋯*Cg*1^iv^	0.93	2.92	3.8418 (17)	173

Symmetry codes: (i) 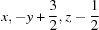; (ii) 
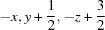
; (iii) 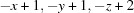; (iv) 
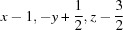
.

**Fig. 1.**
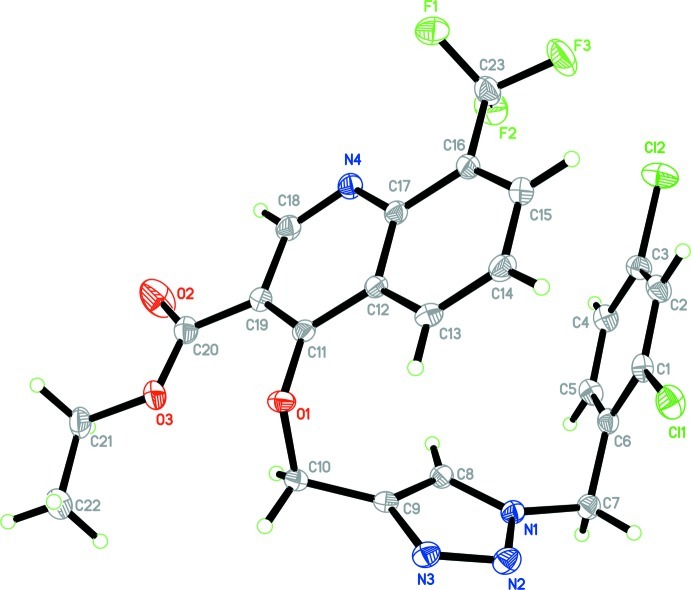
The molecular structure of the title compound, showing 50% probability displacement ellipsoids and the atom-numbering scheme.

**Fig. 2.**
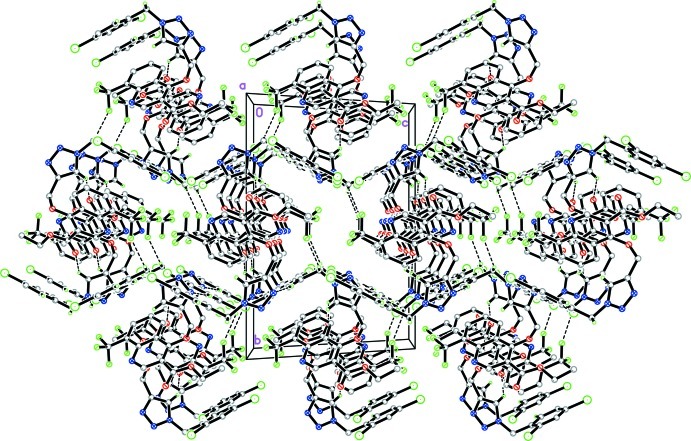
The crystal packing of the title compound, viewed along the a axis. H atoms not involved in the intermolecular interactions (dashed lines) have been omitted for clarity.

## supplementary crystallographic information

### Comment

Quinoline and its derivatives play an important role in medicinal chemistry research. Fluorinated quinolines, in particular CF_3_ substituted quinolines, occupy a significant place in modern medicinal chemistry. Biological studies clearly indicated that the presence of trifluoromethyl group in positions 7 and 8 of the quinoline ring is responsible for the biological activity (Holla *et al.*, 2006; He *et al.*, 2005; Bi *et al.*, 2004). On the other hand, heterocyclic compounds play an important role in an untiring effort aimed at developing new antimicrobial agents with a new mechanism of action. These heterocyclic compounds are well known to possess diverse pharmacological properties, *viz.* antibacterial, antifungal, anti-inflammatory, anticonvulsant, antiviral, antimalarial, antituberculosis, and anticancer effects (Isloor *et al.*, 2000, 2009; Vijesh *et al.*, 2010). In view of this biological importance, we have synthesized the title compound to study its crystal structure.

In the title compound (Fig. 1), the triazole (N1–N3/C8/C9) ring makes dihedral angles of 50.27 (6) and 82.78 (7)° with the quinoline ring system (N4/C11–C19) and the dichloro-substituted benzene ring (C1–C6) respectively. The dihedral angle between the quinoline and the dichloro-substituted benzene ring is 38.17 (4)°. The bond lengths (Allen *et al.*, 1987) and angles are within normal ranges.

In the crystal packing (Fig. 2), the molecules linked *via* intermolecular C5—H5A···N3, C7—H7A···F1 and C8—H8A···O2 hydrogen bonds (Table 1) into a three dimensional network. The crystal is further consolidated by C2—H2A···*Cg*1 interactions (Table 1), involving the triazole ring (N1–N3/C8/C9). Weak π–π interactions are also observed with *Cg*2···*Cg*4 = 3.7037 (7) Å [symmetry code: -*x*, 1 - *y*, 2 - *z*], where *Cg*2 and *Cg*4 are centroids of the pyridine ring (N4/C11/C12/C17/C18/C19) and the benzene ring (C12–C17) respectively.

### Experimental

To a stirred solution of 1-(bromomethyl)-2,4-dichlorobenzene (0.50 g, 0.0020 mol), sodium azide (0.149 g, 0.0022 mol) in aqueous polyethylene glycol (PEG 400) (10 ml, 1:1, *v/v*), ethyl 4-(prop-2-yn-1-yloxy)-8- (trifluoromethyl)quinoline-3-carboxylate (0.711 g, 0.0022 mol), sodium ascorbate (0.435 g, 0.0022 mol), 10 mol of copper iodide were added. The heterogeneous mixture was stirred vigorously overnight. Completion of the reaction was monitored by the TLC. The product was extracted in ethyl acetate and concentrated. The crude product was purified by column chromatography using pet ether and ethyl acetate as eluents. Crystals were grown by slow evaporation of a dilute ethanol solution at room temperature. Yield: 0.35 g, 32.11 %, M. p.: 423–425 K.

### Refinement

All H atoms were positioned geometrically [C–H = 0.93, 0.96 and 0.97 Å] with *U*_iso_(H) = 1.2 or 1.5 *U*_eq_(C). A rotating group model was applied to the methyl group. In the final refinement, four outliers, (0 14 0), (0 13 1), (-3 0 3) and (3 0 0), were omitted.

### Crystal data

**Table:**

C_23_H_17_Cl_2_F_3_N_4_O_3_	*F*(000) = 1072
*M**_r_* = 525.31	*D*_x_ = 1.554 Mg m^−^^3^
Monoclinic, *P*2_1_/*c*	Mo *K*α radiation, λ = 0.71073 Å
Hall symbol: -P 2ybc	Cell parameters from 9559 reflections
*a* = 10.0414 (6) Å	θ = 2.3–32.6°
*b* = 18.3997 (11) Å	µ = 0.35 mm^−^^1^
*c* = 15.5456 (7) Å	*T* = 100 K
β = 128.559 (2)°	Block, colourless
*V* = 2246.0 (2) Å^3^	0.32 × 0.31 × 0.17 mm
*Z* = 4	

### Data collection

**Table:**

Bruker APEX DUO CCD area-detector diffractometer	8173 independent reflections
Radiation source: fine-focus sealed tube	6633 reflections with *I* > 2σ(*I*)
Graphite monochromator	*R*_int_ = 0.028
φ and ω scans	θ_max_ = 32.7°, θ_min_ = 2.0°
Absorption correction: multi-scan (*SADABS*; Bruker, 2009)	*h* = −15→15
*T*_min_ = 0.896, *T*_max_ = 0.942	*k* = −26→27
28920 measured reflections	*l* = −23→22

### Refinement

**Table:**

Refinement on *F*^2^	Primary atom site location: structure-invariant direct methods
Least-squares matrix: full	Secondary atom site location: difference Fourier map
*R*[*F*^2^ > 2σ(*F*^2^)] = 0.035	Hydrogen site location: inferred from neighbouring sites
*wR*(*F*^2^) = 0.102	H-atom parameters constrained
*S* = 1.03	*w* = 1/[σ^2^(*F*_o_^2^) + (0.0562*P*)^2^ + 0.415*P*] where *P* = (*F*_o_^2^ + 2*F*_c_^2^)/3
8173 reflections	(Δ/σ)_max_ = 0.001
317 parameters	Δρ_max_ = 0.49 e Å^−^^3^
0 restraints	Δρ_min_ = −0.27 e Å^−^^3^

### Special details

**Table:**

Experimental. The crystal was placed in the cold stream of an Oxford Cryosystems Cobra open-flow nitrogen cryostat (Cosier & Glazer, 1986) operating at 100.0 (1) K.
Geometry. All e.s.d.'s (except the e.s.d. in the dihedral angle between two l.s. planes) are estimated using the full covariance matrix. The cell e.s.d.'s are taken into account individually in the estimation of e.s.d.'s in distances, angles and torsion angles; correlations between e.s.d.'s in cell parameters are only used when they are defined by crystal symmetry. An approximate (isotropic) treatment of cell e.s.d.'s is used for estimating e.s.d.'s involving l.s. planes.
Refinement. Refinement of *F*^2^ against ALL reflections. The weighted *R*-factor *wR* and goodness of fit *S* are based on *F*^2^, conventional *R*-factors *R* are based on *F*, with *F* set to zero for negative *F*^2^. The threshold expression of *F*^2^ > σ(*F*^2^) is used only for calculating *R*-factors(gt) *etc*. and is not relevant to the choice of reflections for refinement. *R*-factors based on *F*^2^ are statistically about twice as large as those based on *F*, and *R*- factors based on ALL data will be even larger.

### Fractional atomic coordinates and isotropic or equivalent isotropic displacement parameters (Å^2^)

**Table:**

	*x*	*y*	*z*	*U*_iso_*/*U*_eq_	
Cl1	−0.12680 (3)	0.821670 (15)	0.85096 (2)	0.02315 (7)	
Cl2	−0.32434 (4)	0.689263 (18)	0.48063 (2)	0.02946 (7)	
F1	−0.36101 (10)	0.45643 (4)	0.65715 (6)	0.02879 (16)	
F2	−0.25813 (10)	0.52957 (5)	0.60326 (6)	0.03245 (17)	
F3	−0.47656 (10)	0.56112 (5)	0.59016 (6)	0.03603 (19)	
O1	0.32034 (9)	0.56730 (4)	1.13470 (6)	0.01678 (14)	
O2	0.48813 (13)	0.39682 (6)	1.03651 (8)	0.0395 (3)	
O3	0.55544 (11)	0.46603 (4)	1.17639 (7)	0.02473 (17)	
N1	0.29130 (11)	0.78082 (5)	1.00429 (7)	0.01548 (15)	
N2	0.30596 (12)	0.80120 (5)	1.09313 (7)	0.01942 (17)	
N3	0.36803 (12)	0.74507 (5)	1.16052 (7)	0.01799 (16)	
N4	0.01453 (12)	0.47968 (5)	0.81631 (7)	0.01919 (17)	
C1	−0.07552 (13)	0.78849 (6)	0.77012 (8)	0.01699 (18)	
C2	−0.20346 (13)	0.75504 (6)	0.67150 (9)	0.01960 (19)	
H2A	−0.3127	0.7501	0.6499	0.024*	
C3	−0.16378 (13)	0.72921 (6)	0.60602 (8)	0.01965 (19)	
C4	−0.00095 (14)	0.73555 (6)	0.63755 (9)	0.02013 (19)	
H4A	0.0239	0.7175	0.5931	0.024*	
C5	0.12413 (13)	0.76941 (6)	0.73696 (8)	0.01918 (19)	
H5A	0.2335	0.7739	0.7587	0.023*	
C6	0.08973 (13)	0.79674 (5)	0.80482 (8)	0.01678 (17)	
C7	0.22889 (13)	0.83146 (5)	0.91327 (8)	0.01838 (18)	
H7A	0.3221	0.8454	0.9133	0.022*	
H7B	0.1856	0.8750	0.9233	0.022*	
C8	0.34385 (12)	0.71183 (5)	1.01398 (8)	0.01612 (17)	
H8A	0.3464	0.6854	0.9640	0.019*	
C9	0.39297 (12)	0.68906 (5)	1.11442 (8)	0.01461 (16)	
C10	0.45857 (12)	0.61685 (5)	1.16863 (8)	0.01687 (18)	
H10A	0.5263	0.5959	1.1500	0.020*	
H10B	0.5318	0.6231	1.2478	0.020*	
C11	0.22978 (12)	0.53905 (5)	1.03163 (8)	0.01451 (16)	
C12	0.05997 (12)	0.56544 (5)	0.95288 (8)	0.01491 (17)	
C13	−0.00764 (13)	0.62046 (5)	0.97944 (8)	0.01711 (18)	
H13A	0.0597	0.6409	1.0494	0.021*	
C14	−0.17216 (13)	0.64365 (5)	0.90227 (9)	0.01925 (19)	
H14A	−0.2161	0.6797	0.9202	0.023*	
C15	−0.27516 (14)	0.61315 (6)	0.79582 (9)	0.01969 (19)	
H15A	−0.3863	0.6295	0.7439	0.024*	
C16	−0.21298 (13)	0.55957 (5)	0.76808 (8)	0.01790 (18)	
C17	−0.04291 (13)	0.53391 (5)	0.84626 (8)	0.01585 (17)	
C18	0.17098 (14)	0.45736 (6)	0.89147 (9)	0.01910 (19)	
H18A	0.2102	0.4206	0.8714	0.023*	
C19	0.28671 (13)	0.48409 (5)	1.00108 (8)	0.01653 (17)	
C20	0.45265 (14)	0.44489 (6)	1.07212 (9)	0.01967 (19)	
C21	0.71534 (15)	0.42565 (6)	1.24926 (10)	0.0268 (2)	
H21A	0.7758	0.4249	1.2192	0.032*	
H21B	0.6929	0.3759	1.2575	0.032*	
C22	0.81915 (19)	0.46389 (8)	1.35829 (12)	0.0428 (4)	
H22A	0.9215	0.4368	1.4103	0.064*	
H22B	0.7543	0.4676	1.3843	0.064*	
H22C	0.8481	0.5117	1.3500	0.064*	
C23	−0.32547 (15)	0.52651 (6)	0.65532 (9)	0.0236 (2)	

### Atomic displacement parameters (Å^2^)

**Table:**

	*U*^11^	*U*^22^	*U*^33^	*U*^12^	*U*^13^	*U*^23^
Cl1	0.02094 (12)	0.02780 (13)	0.02435 (13)	0.00553 (9)	0.01591 (10)	0.00119 (9)
Cl2	0.02577 (13)	0.03967 (16)	0.02093 (13)	−0.01115 (11)	0.01358 (11)	−0.00591 (10)
F1	0.0312 (4)	0.0247 (3)	0.0217 (3)	−0.0053 (3)	0.0121 (3)	−0.0087 (3)
F2	0.0371 (4)	0.0409 (4)	0.0170 (3)	0.0021 (3)	0.0157 (3)	0.0006 (3)
F3	0.0250 (4)	0.0398 (4)	0.0187 (3)	0.0109 (3)	0.0015 (3)	−0.0020 (3)
O1	0.0178 (3)	0.0181 (3)	0.0134 (3)	−0.0053 (3)	0.0091 (3)	−0.0027 (2)
O2	0.0297 (5)	0.0477 (6)	0.0332 (5)	0.0175 (4)	0.0157 (4)	−0.0051 (4)
O3	0.0222 (4)	0.0196 (4)	0.0207 (4)	0.0056 (3)	0.0076 (3)	0.0040 (3)
N1	0.0157 (3)	0.0153 (4)	0.0159 (4)	−0.0003 (3)	0.0100 (3)	−0.0006 (3)
N2	0.0237 (4)	0.0178 (4)	0.0196 (4)	−0.0005 (3)	0.0149 (4)	−0.0022 (3)
N3	0.0205 (4)	0.0170 (4)	0.0184 (4)	−0.0016 (3)	0.0130 (3)	−0.0020 (3)
N4	0.0222 (4)	0.0165 (4)	0.0167 (4)	0.0015 (3)	0.0110 (3)	−0.0019 (3)
C1	0.0175 (4)	0.0174 (4)	0.0181 (4)	0.0041 (3)	0.0121 (4)	0.0048 (3)
C2	0.0152 (4)	0.0234 (5)	0.0197 (4)	0.0010 (3)	0.0106 (4)	0.0039 (4)
C3	0.0186 (4)	0.0213 (5)	0.0166 (4)	−0.0021 (3)	0.0098 (4)	0.0021 (3)
C4	0.0212 (5)	0.0234 (5)	0.0188 (4)	0.0014 (4)	0.0139 (4)	0.0029 (4)
C5	0.0163 (4)	0.0230 (5)	0.0193 (4)	0.0007 (3)	0.0117 (4)	0.0041 (4)
C6	0.0160 (4)	0.0161 (4)	0.0176 (4)	0.0019 (3)	0.0101 (4)	0.0042 (3)
C7	0.0185 (4)	0.0160 (4)	0.0194 (4)	−0.0003 (3)	0.0112 (4)	0.0030 (3)
C8	0.0164 (4)	0.0156 (4)	0.0160 (4)	0.0011 (3)	0.0099 (3)	−0.0009 (3)
C9	0.0129 (4)	0.0152 (4)	0.0143 (4)	−0.0024 (3)	0.0078 (3)	−0.0019 (3)
C10	0.0142 (4)	0.0165 (4)	0.0148 (4)	−0.0024 (3)	0.0066 (3)	−0.0003 (3)
C11	0.0170 (4)	0.0128 (4)	0.0134 (4)	−0.0018 (3)	0.0094 (3)	−0.0002 (3)
C12	0.0169 (4)	0.0123 (4)	0.0145 (4)	−0.0010 (3)	0.0092 (3)	−0.0005 (3)
C13	0.0191 (4)	0.0147 (4)	0.0179 (4)	−0.0025 (3)	0.0117 (4)	−0.0035 (3)
C14	0.0209 (5)	0.0145 (4)	0.0236 (5)	0.0003 (3)	0.0145 (4)	−0.0021 (3)
C15	0.0185 (4)	0.0167 (4)	0.0201 (5)	0.0019 (3)	0.0101 (4)	0.0013 (3)
C16	0.0187 (4)	0.0156 (4)	0.0142 (4)	0.0006 (3)	0.0077 (4)	−0.0001 (3)
C17	0.0185 (4)	0.0136 (4)	0.0144 (4)	−0.0001 (3)	0.0097 (3)	−0.0004 (3)
C18	0.0225 (5)	0.0159 (4)	0.0193 (4)	0.0011 (3)	0.0133 (4)	−0.0022 (3)
C19	0.0172 (4)	0.0149 (4)	0.0172 (4)	0.0006 (3)	0.0105 (4)	0.0009 (3)
C20	0.0189 (4)	0.0192 (4)	0.0215 (5)	0.0021 (3)	0.0129 (4)	0.0031 (3)
C21	0.0202 (5)	0.0219 (5)	0.0277 (5)	0.0048 (4)	0.0099 (4)	0.0102 (4)
C22	0.0331 (7)	0.0265 (6)	0.0304 (7)	0.0013 (5)	0.0009 (5)	0.0054 (5)
C23	0.0224 (5)	0.0242 (5)	0.0154 (4)	0.0035 (4)	0.0075 (4)	−0.0002 (4)

### Geometric parameters (Å, º)

**Table:**

Cl1—C1	1.7387 (11)	C7—H7B	0.9700
Cl2—C3	1.7383 (11)	C8—C9	1.3772 (14)
F1—C23	1.3429 (14)	C8—H8A	0.9300
F2—C23	1.3408 (15)	C9—C10	1.4891 (14)
F3—C23	1.3482 (13)	C10—H10A	0.9700
O1—C11	1.3594 (11)	C10—H10B	0.9700
O1—C10	1.4571 (12)	C11—C19	1.3833 (14)
O2—C20	1.2089 (14)	C11—C12	1.4282 (14)
O3—C20	1.3260 (14)	C12—C13	1.4161 (14)
O3—C21	1.4649 (13)	C12—C17	1.4201 (13)
N1—C8	1.3466 (13)	C13—C14	1.3711 (15)
N1—N2	1.3477 (12)	C13—H13A	0.9300
N1—C7	1.4689 (13)	C14—C15	1.4105 (15)
N2—N3	1.3186 (12)	C14—H14A	0.9300
N3—C9	1.3646 (13)	C15—C16	1.3724 (15)
N4—C18	1.3091 (14)	C15—H15A	0.9300
N4—C17	1.3710 (13)	C16—C17	1.4244 (14)
C1—C2	1.3873 (15)	C16—C23	1.5001 (15)
C1—C6	1.3970 (14)	C18—C19	1.4239 (14)
C2—C3	1.3878 (15)	C18—H18A	0.9300
C2—H2A	0.9300	C19—C20	1.4908 (14)
C3—C4	1.3886 (15)	C21—C22	1.5006 (19)
C4—C5	1.3908 (15)	C21—H21A	0.9700
C4—H4A	0.9300	C21—H21B	0.9700
C5—C6	1.3925 (15)	C22—H22A	0.9600
C5—H5A	0.9300	C22—H22B	0.9600
C6—C7	1.5054 (14)	C22—H22C	0.9600
C7—H7A	0.9700		
			
C11—O1—C10	116.90 (8)	C19—C11—C12	118.84 (9)
C20—O3—C21	116.06 (9)	C13—C12—C17	120.01 (9)
C8—N1—N2	111.27 (8)	C13—C12—C11	121.76 (9)
C8—N1—C7	127.43 (9)	C17—C12—C11	118.21 (9)
N2—N1—C7	121.28 (8)	C14—C13—C12	120.17 (9)
N3—N2—N1	106.97 (8)	C14—C13—H13A	119.9
N2—N3—C9	108.95 (8)	C12—C13—H13A	119.9
C18—N4—C17	116.75 (9)	C13—C14—C15	120.34 (10)
C2—C1—C6	122.25 (10)	C13—C14—H14A	119.8
C2—C1—Cl1	117.85 (8)	C15—C14—H14A	119.8
C6—C1—Cl1	119.89 (8)	C16—C15—C14	120.67 (10)
C1—C2—C3	118.10 (10)	C16—C15—H15A	119.7
C1—C2—H2A	120.9	C14—C15—H15A	119.7
C3—C2—H2A	120.9	C15—C16—C17	120.55 (9)
C2—C3—C4	121.71 (10)	C15—C16—C23	119.98 (9)
C2—C3—Cl2	118.53 (8)	C17—C16—C23	119.45 (9)
C4—C3—Cl2	119.75 (9)	N4—C17—C12	122.67 (9)
C3—C4—C5	118.61 (10)	N4—C17—C16	119.07 (9)
C3—C4—H4A	120.7	C12—C17—C16	118.26 (9)
C5—C4—H4A	120.7	N4—C18—C19	126.15 (10)
C4—C5—C6	121.66 (10)	N4—C18—H18A	116.9
C4—C5—H5A	119.2	C19—C18—H18A	116.9
C6—C5—H5A	119.2	C11—C19—C18	117.37 (9)
C5—C6—C1	117.65 (9)	C11—C19—C20	127.57 (9)
C5—C6—C7	120.41 (9)	C18—C19—C20	114.90 (9)
C1—C6—C7	121.90 (10)	O2—C20—O3	123.18 (10)
N1—C7—C6	110.51 (8)	O2—C20—C19	121.81 (10)
N1—C7—H7A	109.5	O3—C20—C19	115.00 (9)
C6—C7—H7A	109.5	O3—C21—C22	106.89 (11)
N1—C7—H7B	109.5	O3—C21—H21A	110.3
C6—C7—H7B	109.5	C22—C21—H21A	110.3
H7A—C7—H7B	108.1	O3—C21—H21B	110.3
N1—C8—C9	104.60 (9)	C22—C21—H21B	110.3
N1—C8—H8A	127.7	H21A—C21—H21B	108.6
C9—C8—H8A	127.7	C21—C22—H22A	109.5
N3—C9—C8	108.21 (9)	C21—C22—H22B	109.5
N3—C9—C10	122.53 (9)	H22A—C22—H22B	109.5
C8—C9—C10	129.24 (9)	C21—C22—H22C	109.5
O1—C10—C9	111.58 (8)	H22A—C22—H22C	109.5
O1—C10—H10A	109.3	H22B—C22—H22C	109.5
C9—C10—H10A	109.3	F2—C23—F1	107.08 (9)
O1—C10—H10B	109.3	F2—C23—F3	106.34 (9)
C9—C10—H10B	109.3	F1—C23—F3	106.27 (10)
H10A—C10—H10B	108.0	F2—C23—C16	113.11 (10)
O1—C11—C19	124.63 (9)	F1—C23—C16	112.41 (9)
O1—C11—C12	116.38 (9)	F3—C23—C16	111.19 (9)
			
C8—N1—N2—N3	−0.11 (11)	C11—C12—C13—C14	−178.94 (10)
C7—N1—N2—N3	−178.65 (9)	C12—C13—C14—C15	−0.22 (16)
N1—N2—N3—C9	0.08 (11)	C13—C14—C15—C16	0.47 (17)
C6—C1—C2—C3	−0.03 (15)	C14—C15—C16—C17	−0.20 (17)
Cl1—C1—C2—C3	179.26 (8)	C14—C15—C16—C23	178.60 (10)
C1—C2—C3—C4	0.65 (16)	C18—N4—C17—C12	0.28 (15)
C1—C2—C3—Cl2	−178.39 (8)	C18—N4—C17—C16	−179.17 (10)
C2—C3—C4—C5	−0.66 (16)	C13—C12—C17—N4	−178.92 (10)
Cl2—C3—C4—C5	178.36 (8)	C11—C12—C17—N4	−0.22 (15)
C3—C4—C5—C6	0.05 (16)	C13—C12—C17—C16	0.53 (15)
C4—C5—C6—C1	0.53 (15)	C11—C12—C17—C16	179.23 (9)
C4—C5—C6—C7	178.48 (9)	C15—C16—C17—N4	179.18 (10)
C2—C1—C6—C5	−0.54 (15)	C23—C16—C17—N4	0.38 (15)
Cl1—C1—C6—C5	−179.82 (8)	C15—C16—C17—C12	−0.29 (15)
C2—C1—C6—C7	−178.46 (9)	C23—C16—C17—C12	−179.10 (10)
Cl1—C1—C6—C7	2.27 (13)	C17—N4—C18—C19	−0.02 (17)
C8—N1—C7—C6	51.57 (13)	O1—C11—C19—C18	175.75 (9)
N2—N1—C7—C6	−130.14 (10)	C12—C11—C19—C18	0.33 (14)
C5—C6—C7—N1	−101.78 (11)	O1—C11—C19—C20	0.67 (17)
C1—C6—C7—N1	76.08 (12)	C12—C11—C19—C20	−174.75 (10)
N2—N1—C8—C9	0.08 (11)	N4—C18—C19—C11	−0.28 (17)
C7—N1—C8—C9	178.52 (9)	N4—C18—C19—C20	175.42 (10)
N2—N3—C9—C8	−0.03 (11)	C21—O3—C20—O2	−2.25 (17)
N2—N3—C9—C10	−178.60 (9)	C21—O3—C20—C19	176.76 (9)
N1—C8—C9—N3	−0.03 (11)	C11—C19—C20—O2	179.10 (12)
N1—C8—C9—C10	178.41 (9)	C18—C19—C20—O2	3.91 (16)
C11—O1—C10—C9	74.10 (11)	C11—C19—C20—O3	0.08 (16)
N3—C9—C10—O1	91.43 (11)	C18—C19—C20—O3	−175.10 (9)
C8—C9—C10—O1	−86.82 (13)	C20—O3—C21—C22	173.46 (11)
C10—O1—C11—C19	75.83 (12)	C15—C16—C23—F2	125.51 (11)
C10—O1—C11—C12	−108.65 (10)	C17—C16—C23—F2	−55.68 (13)
O1—C11—C12—C13	2.78 (14)	C15—C16—C23—F1	−113.06 (11)
C19—C11—C12—C13	178.58 (9)	C17—C16—C23—F1	65.75 (14)
O1—C11—C12—C17	−175.90 (8)	C15—C16—C23—F3	5.93 (16)
C19—C11—C12—C17	−0.10 (14)	C17—C16—C23—F3	−175.25 (10)
C17—C12—C13—C14	−0.28 (15)		

### Hydrogen-bond geometry (Å, º)

Cg1 is the centroid of the N1–N3/C8/C9 triazole ring

**Table:**

*D*—H···*A*	*D*—H	H···*A*	*D*···*A*	*D*—H···*A*
C5—H5*A*···N3^i^	0.93	2.62	3.3330 (14)	134
C7—H7*A*···F1^ii^	0.97	2.46	3.1712 (15)	130
C8—H8*A*···O2^iii^	0.93	2.25	3.0183 (18)	139
C2—H2*A*···*Cg*1^iv^	0.93	2.92	3.8418 (17)	173

Symmetry codes: (i) *x*, −*y*+3/2, *z*−1/2; (ii) −*x*, *y*+1/2, −*z*+3/2; (iii) −*x*+1, −*y*+1, −*z*+2; (iv) *x*−1, −*y*+1/2, *z*−3/2.
